# Combined-modality hypofractionated radiotherapy for elderly patients with glioblastoma: setting a new standard

**DOI:** 10.4155/fsoa-2017-0050

**Published:** 2017-06-15

**Authors:** Balamurugan A Vellayappan, Lia M Halasz, Jonathan PS Knisely, Eric L Chang, Simon S Lo

**Affiliations:** 1 Department of Radiation Oncology, National University Cancer Institute Singapore, National University Hospital, Singapore; 2 Department of Radiation Oncology, University of Washington School of Medicine, Seattle, WA, USA; 3 Department of Radiation Medicine, Northwell Health & Hofstra Northwell School of Medicine, Lake Success, NY, USA; 4 Department of Radiation Oncology, University of Southern California, Keck School of Medicine, Los Angeles, CA, USA

**Keywords:** chemotherapy, geriatrics, glioblastoma, radiotherapy, trials

Glioblastoma (GBM) is the most common malignant primary brain tumour. It disproportionately affects the older segment of the population, as approximately half of the patients with GBM are above 65 years [1,2]. Moreover, the aging of the baby boom generation foreshadows a ‘silver tsunami’ of GBM [1].

Overlapping problems complicate management decisions for elderly patients, whether or not they have GBM. These may include multiple comorbidities and poor physiologic reserves, polypharmacy, limited mobility, cognitive decline, and social and financial vulnerability [3]. In addition, survival among elderly patients with GBM has been consistently shorter than among younger patients, with a population based median survival of approximately 6 months (m) [4]. It is unclear if this is due to more aggressive tumor biology [5], the use of less intense treatment approaches [3] or increased susceptibility to treatment toxicities [6].

The standard of care for GBM management for patients below 70 years of age, has been defined by the EORTC 26981/22981 NCIC-CE3 trial [7], fondly referred to as the ‘Stupp regimen’. This international, randomized controlled trial compared concurrent and adjuvant temozolomide (TMZ) with conventionally fractionated radiation therapy (RT; 60 Gy/30 fractions) to RT alone (60 Gy/30 fractions). The combined modality therapy (CMT) group had improved overall survival (OS) compared with the RT alone group (hazard ratio [HR]: 0.63 95%; CI: 0.52–0.75; p < 0.001). The 2-year OS were 27 and 10%, respectively. The presence of O(6)-methylguanine-DNA methyltransferase (MGMT) methylation greatly accentuated the survival benefit with TMZ. The 5-year analysis [8] was published in 2009 and investigated if the benefit of TMZ was seen in older patients. Patients between 60 and 70 years of age comprised 30% of the participants. The median survival for this subgroup was lower with CMT (10.9 vs 11.8 m). An unplanned subgroup analysis showed that survival differences for the 65–70-years old cohort failed to reach statistical significance (HR: 0.78; 95% CI: 0.5–1.24; p = 0.29) [2]. This suggested that older patients derived less benefit from CMT, although it had to be interpreted with caution due to the small patient numbers.

Many elderly patients with GBM seen in daily clinical practice do not qualify for the ‘Stupp regimen’ because of their age. Unfortunately, elderly patients have been excluded from many randomized clinical trials, and hence there is a lack of consensus on how this group should be managed – especially with regard to CMT.

Across various cancer types [9–11], hypofractionated radiotherapy regimens have been increasingly adopted. Hypofractionated regimens reduce resource utilization for the healthcare system, potentially reduce costs and reduce the time spent by the patient commuting to and at the hospital. For instance, for a patient with an expected survival of 7 m, a regimen that is 3 weeks shorter would free up 10% of their remaining lifetime spent with hospital visits. Roa *et al*. [12] demonstrated the noninferiority of 40 Gy/15 fractions compared with 60 Gy/30 fractions for patients with GBM aged 60 years and over with Karnofsky Performance status (KPS) ≥50. Standard fractionation and hypofractionation provided OS of 5.1 and 5.6 m, respectively (p = 0.57). Moreover, fewer patients in the hypofractionated arm stopped treatment prematurely (10 vs 25%), suggesting better tolerability. However, widespread adoption was blunted as the reported survival was shorter than expected, and many felt the included patients were not representative of everyday practice. More recently, shorter regimens such as 25 Gy/5 fractions [13] and 34 Gy/10 fractions [14] have shown to be equally effective in elderly and/or frail patients. However, it has to be noted that the definition of elderly has varied among these trials from above 60 [14], 65 [15] and 70 years [16].

Compared with the ‘Stupp regimen’, hypofractionated RT regimens have been largely regarded as ‘palliative’ treatments. Hence, the aim of such treatments is to provide reasonable longevity as well as palliation without increasing treatment-related toxicity. To that end, many favored single-modality therapy as an approach with less toxicity. For example, the Nordic trial [14] compared three single modality treatments (60 Gy/30 fractions RT alone, TMZ alone [200 mg/m^2^ for 5 days every 28 days] and 34 Gy/10 fractions hypofractionated RT alone) in elderly patients with GBM. In this trial, patients with TMZ alone or hypofractionated RT alone had better outcomes than those treated with 60 Gy.

In addition, regimens with fraction sizes larger than 2 Gy are seldom combined with concurrent chemotherapy for the same concerns of worse acute and late toxicities. However, late neurological toxicity is unlikely to clinically manifest in this group of patients with a short lifespan. Cao *et al*. [17] performed a retrospective study where patients >60 years (n = 112) were treated with hypofractionated RT (40 Gy/15 fractions) with concurrent TMZ. Although a survival benefit was not demonstrable (median OS 6.9 vs 9.3 months for RT alone), only 9% of patients (from the combined modality arm) reported grade 3 or 4 hematological toxicity. Minniti *et al*. [18] conducted a prospective Phase II trial involving 71 patients >70 years who were treated with hypofractionated RT (40 Gy/15 fractions) with concurrent TMZ and adjuvant TMZ (12 cycles). Patients from this cohort achieved a median survival of 12.4 months with a progression-free survival of 6 months. This study showed that this regimen is well-tolerated with only 8% of patients having treatment interruption. Grade 3–4 toxicities occurred in 22% of patients, most of which were hematological.

Given the controversy over optimal treatment of elderly patients, Perry *et al*. are to be commended for designing and completing this international, randomized controlled trial (NCIC-CE6/EORTC 26062–22061) [19]. From 2007 to 2013, 562 patients aged 65 years and above, with Eastern Cooperative Oncology Group (ECOG) performance status ≥2 were randomized to hypofractionated RT 40 Gy/15 fractions with concurrent and adjuvant TMZ (12 cycles) versus RT alone (40 Gy/15 fractions). The arms were equally balanced in number and baseline characteristics. The median age was 73 years (range: 65–90 years), with two-thirds aged over 70 years. It has to be stressed that only patients deemed unsuitable for ‘Stupp regimen’ therapy were eligible for this study. Overall, 68.3% of patients underwent a partial or complete resection, and the remainder had biopsies only. The median OS was longer in the CMT arm (9.3 vs 7.6 months; HR: 0.67; 95% CI: 0.56–0.80; p < 0.001). The median PFS was also longer (5.3 vs 3.9 months; HR: 0.50; 95% CI: 0.41–0.60; p < 0.001).

MGMT status was available in 62.9% of participants, and promoter methylation was found in 46.6%. Among these patients, median OS was much improved with CMT (13.5 vs 7.7 months; HR: 0.53; 95% CI: 0.38–0.73; p < 0.001). An exploratory analysis suggested that combined modality in the MGMT methylated subgroup was associated with a persistent survival advantage at 24 months. In the unmethylated subgroup, median OS improvement was still clinically meaningful but just short of statistical significance (10.0 vs 7.9 months; HR: 0.75; 95% CI: 0.56–1.01; p = 0.055). More importantly, although toxicity (mostly hematological and gastrointestinal) was worse in the CMT group, quality-of-life scores (measured through QLQ-C30 and QLQ-BN20) were similar in both arms. Treatment tolerability and adherence were high in both arms. More than 97% of patients completed radiotherapy and the median number of adjuvant TMZ cycles was five.

Patients were stratified at randomization according to their age groups: 65–70 years, 71–75 years, 75 years and above. It was surprising that the treatment effect was more pronounced in the older age groups, although this failed to reach statistical significance (p = 0.06 for interaction). This may be due to a selection bias, as only patients, including the 65–70 years subgroups, who were deemed unsuitable for ‘Stupp regimen’ were eligible for this study. As such, this group may have inherently worse outcomes from the outset.

40% of the patients received some type of systemic therapy on progression, and the proportion was equal across the arms. More patients received salvage TMZ from the RT only arm. Patients from the CMT arm received agents other than TMZ, such as lomustine or bevacizumab. It is nearly impossible to determine whether these treatments were associated with longer survival, or whether the longer survival provided more opportunity for these patients to receive salvage systemic therapy.

It is clear that this study will change our practice for management of elderly patients with GBM. The treatment regimen used in this study is effective and tolerable and offers a meaningful benefit to older patients. However, there are still many unanswered questions:

- How does 40 Gy/15 fractions and TMZ compare to 60 Gy/30 fractions + TMZ?
- As in the ‘Stupp regimen’, we are still unsure if most of the benefit comes from the concurrent portion of TMZ or the adjuvant portion of TMZ – clarifying this may guide us in choosing the ‘minimum required treatment’ for patients unable to tolerate both concurrent and adjuvant phases;
- Can we do away with RT altogether for patients with MGMT methylation? [14,15]
- Can we combine TMZ with other hypofractionated regimens such as 34 Gy/10 or 25 Gy/5 fractions?

Undoubtedly, further trials will be needed to answer these questions and guide our practice.

Last, age is just a number and can be regarded as a ‘soft’ factor when deciding therapy. Age has been emphasized in clinical trials, and is included in many prognostic scoring systems, due to its objectivity and ubiquity. Tumor molecular profiling, physiological age and performance status, may be more important to determine which patients may benefit from more aggressive treatment.
